# A rapid access to addiction medicine clinic facilitates treatment of substance use disorder and reduces substance use

**DOI:** 10.1186/s13011-019-0250-1

**Published:** 2020-01-13

**Authors:** David Wiercigroch, Hasan Sheikh, Jennifer Hulme

**Affiliations:** 1 Faculty of Medicine, University of Toronto, 1 King’s College Cir, Toronto, Ontario M5S 1A8 Canada; 2 Global Health Emergency Medicine, Toronto General Hospital, 200 Elizabeth Street, 12NU-1320, Toronto, ON M5G 2C4 Canada; 3 Department of Family and Community Medicine, University of Toronto, 500 University Avenue, 5th Floor, Toronto, Ontario M5G 1V7 Canada; 4 Emergency Department, University Health Network, 200 Elizabeth Street, R. Fraser Elliott Building, Ground Floor, Room 480, Toronto, ON M5G 2C4 Canada

**Keywords:** Low-barrier, alcohol, Opioid, Buprenorphine, Addiction, Outpatient, RAAM, substance use

## Abstract

**Background:**

Substance use is prevalent in Canada, yet treatment is inaccessible. The Rapid Access to Addiction Medicine (RAAM) clinic opened at the University Health Network (UHN) in January 2018 as part of a larger network of addictions clinics in Toronto, Ontario, to enable timely, low barrier access to medical treatment for substance use disorder (SUD). Patients attend on a walk-in basis without requiring an appointment or referral. We describe the RAAM clinic model, including referral patterns, patient demographics and substance use patterns. Secondary outcomes include retention in treatment and changes in both self-reported and objective substance use.

**Methods:**

The Electronic Medical Record at the clinic was reviewed for the first 26 weeks of the clinic’s operation. We identified SUD diagnoses, referral source, medications prescribed, retention in care and self-reported substance use.

**Results:**

The clinic saw 64 unique patients: 66% had alcohol use disorder (AUD), 39% had opiate use disorder (OUD) and 20% had stimulant use disorder. Fifty-five percent of patients were referred from primary care providers, 30% from the emergency department and 11% from withdrawal management services. Forty-two percent remained on-going patients, 23% were discharged to other care and 34% were lost to follow-up. Gabapentin (39%), naltrexone (39%), and acamprosate (15%) were most frequently prescribed for AUD. Patients with AUD reported a significant decrease in alcohol consumption at their most recent visit. Most patients (65%) with OUD were prescribed buprenorphine, and most patients with OUD (65%) had a negative urine screen at their most recent visit.

**Conclusion:**

The RAAM model provides low-barrier, accessible outpatient care for patients with substance use disorder and facilitates the prescription of evidence-based pharmacotherapy for AUD and OUD. Patients referred by their primary care physician and the emergency department demonstrated a reduction in median alcohol consumption and high rates of opioid abstinence.

## Introduction

About 1 in 3 Canadian adults meet the criteria for substance use disorder (SUD) during their lifetime as per the Statistics Canada Community Health Survey [1]. Substance use disorder is a term defined in the DSM V which combines criteria for substance abuse and dependence [2]. Among several diagnosable SUDs, alcohol use disorder (AUD) is the most common in Canada, 18.2% of adults meet criteria during their lifetime [1]. Opioid use disorder (OUD) is also a growing public health concern; opioid medications, which carry a 5.5% risk of addiction, are still widely prescribed [3] and opioid-related deaths continue to rise in Canada [4]. SUDs are also associated with utilization of the emergency department (ED): over the past decade ED visits attributable to alcohol have steadily increased and currently opioid poisonings result in approximately 7 ED visits in Ontario every day, and more than 13 hospitalizations a day across Canada [5]. Addiction treatment is a public health pillar and a cost-effective intervention to reduce substance use [6, 7], yet in developed countries, unmet needs of substance users, particularly alcohol users, are universally high – and as high as 78.1% in Ontario [8]. The increasing health burden of substance use in Canada indicates that the current approach to SUD treatment does not sufficiently meet the needs of the adult population and greater access to appropriate treatment is required.

Current models of care do not serve the substance-using population adequately. Withdrawal management services (WMS) are often the first point of contact for individuals seeking treatment for substance use [9] but these are non-medical detoxification centers which do not facilitate access to evidence-based pharmacotherapy concurrently as part of effective treatment [10, 11]. While residential treatment programs for substance use combine medical and psychosocial modalities, they often suffer from attrition given the long wait times [12–14]. Substance users also report poorer access to primary care [13, 14] as well as increased stigma in the waiting room, and when interacting with health care providers [13]. Primary care physicians lack the expertise to properly treat substance use [13]; substance users frequently use emergency medical services to address their primary care needs [14]. Alcohol-related diagnoses such as intoxication, withdrawal and dependence, are a significant reason for frequent ED visits [15]. A flexible model of care is required which connects patients from across these traditional care pathways and facilitates rapid access to medical treatment of substance use disorders.

Substance use disorders are chronic conditions that require longitudinal care beyond what can be offered by short-term programs and acute care services. The National Institute of Drug Abuse recommends expert-informed decision support (i.e. education, expert consultation, standardized assessment tools and treatment algorithms) for primary care providers in the treatment of substance use disorders similar to chronic care models used in cardiology and diabetes care [16]. The chronic care model is a well-established framework for comprehensive, patient-centered chronic disease management that supports increased functional and clinical outcomes [17]. Primary care should be at the center of a chronic care model, however for SUDs further supports are required [16]. Primary care physicians express a need for access to expert consultation when prescribing pharmacotherapies for alcohol and opioid use disorders [18]. Evaluation of new models of care that lower barriers to addictions care and therefore better serve patients with SUDs need to be actively disseminated to facilitate knowledge exchange and inform policy and practice.

This paper reports on the outcomes of a new rapid access to addiction medicine (RAAM) clinic affiliated with the emergency department (ED), family health team (FHT), and withdrawal management services (WMS) in the first 26 weeks of operation. This RAAM clinic is part of a larger network of clinics under the Mentoring, Education, and Clinical Tools for Addiction: Primary Care – Hospital Integration (META:PHI) project [19]. The RAAM clinics help facilitate timely medical management of SUD by eliminating barriers to receiving care: no appointment or referral is required to attend thus allowing patients to be seen within a few days when they are motivated to seek treatment. The RAAM clinics also connect patients with additional counselling resources and community programs.

## Methods

### Study aim

Our primary objective was to describe the RAAM clinic model of care, including referral patterns, patient demographics and substance use disorders. Secondary objectives included the rate of prescription of evidence-based pharmacotherapy for AUD and OUD and treatment outcomes over the study period, including changes in both self-reported and objective substance use, as well as retention in treatment.

Success of the model was evaluated through retention in care, reduction in substance use and provision of evidence-based pharmacotherapy for SUD. Retention in care indicated that patients were receiving treatment for SUD. Previous studies have reported retention rates between 30 and 40% [20, 21]. Given the short period of study, the retention rate was expected to be higher since attrition occurs over time [20, 21] and patients who were discharged to another medical provider were still considered retained in care. A reduction of substance use was considered a successful outcome since decreased substance use is correlated with less severe substance use disorder [22]. Substance use was measured via total drinks and number of abstinent days within the past week. The maximum number of drinks within a day over the past week was used as a measure of binge drinking behavior. Cessation of opioid use was measured via negative urine screen for opioids except buprenorphine if prescribed as therapy. The need for medical therapy for substance use disorder as a component of successful treatment is well established [10, 11] justifying provision of pharmacotherapy for SUD as an appropriate outcome of success.

### Study description

We reviewed the Electronic Medical Record at the UHN RAAM Clinic for the first 26 weeks since the clinic opened, from 01/2018 to the end of 06/2018 inclusively. We tracked patient demographic data including gender, age, and referral source. We also tracked substance use disorder diagnosis and prescribed medications.

Patients were classified based on their status as either an on-going patient, discharged, or lost to follow-up. On-going patients had an active prescription for an addiction-specific medication or notes in the electronic record that indicated on-going care and had attended the clinic at least twice. Discharged patients had their care transferred to another physician or started a residential treatment program where medication for substance use was prescribed. We considered all other patients lost to follow-up. Patients who were discharged or remained on-going patients were considered retained in treatment for substance use disorder.

For patients with AUD, we obtained self-reported measures of alcohol intake using standardized drinks as outlined in Canada’s Low-Risk Alcohol Drinking Guideline [23]. At each visit, patients self-reported the total number of drinks in the past week, the maximum number of drinks in a single day within the past week, and the total number of abstinent days in the past week. For patients with OUD, urine drug screening was performed at each visit. The urine drug screening kit tested for: MDMA, cocaine, amphetamines, opiates, heroin, fentanyl, methadone, EDPP (a methadone metabolite), buprenorphine, and tetrahydrocannabinol.

To assess the prescription of evidence-based pharmacotherapy for alcohol and opioid use disorders, we reported the total number of medications prescribed and the number of patients who received prescriptions. Medications prescribed were only reported for on-going and discharged patients given the lack of data on patients lost to follow-up.

To assess alcohol intake, we compared self-reported responses during the initial visit with responses at the most recent visit. This outcome was assessed using a lost observation carry over method whereby if patients were lost to follow-up, measures of alcohol intake reported at the initial visit were used as the most recent measure of intake.

The University of Toronto Research Ethics Board determined this project to be a quality improvement project that was exempt and not considered human subjects research. Chart reviews were retrospective and patient data was de-identified.

### Targeted program evaluated

The rapid access to addiction medicine (RAAM) outpatient addictions clinic opened in January 2018 at one of the two large, academic teaching hospitals at the University Health Network (UHN). Under the META:PHI project [19], this was only one of 46 RAAM clinics open across the province of Ontario, 9 of which were distributed across the city of Toronto [19]. The model was established to provide timely, low-barrier access to treatment for substance use disorder. Patients did not require an appointment or formal referral to attend. Patients were seen on a walk-in basis and were connected to a RAAM clinic via self-referral, peer-referral, or referral by primary care, the emergency department, or withdrawal management services. The META:PHI network also housed a resource hub for clinicians on substance use disorders in the ED and primary care settings including withdrawal scales, pre-printed orders for alcohol and opioid withdrawal, treatment protocols and strategies for brief counselling among other resources [19].

The UHN RAAM Clinic was closely integrated with the Toronto Western Family Health Team (FHT), defined as a primary care organization whose programs and services are geared towards the population they serve and can include a variety of health care professionals such as family physicians, nurse practitioners, registered nurses, social workers, and dieticians [24]. The RAAM Clinic electronic medical record was connected to the FHT, allowing for shared record-keeping and electronic communication. Furthermore, the clinic was in one of the UHN hospitals near the ED and was easily accessible by patients who were referred from the emergency department at the other UHN hospital site via a free shuttle bus. Finally, the clinic was within walking distance of local withdrawal management services.

The multidisciplinary team at the RAAM clinic included a rotating staff physician and a nurse with experience in addiction treatment and counselling. The rotating staff physician team included family medicine, psychiatry and emergency medicine-trained physicians who had additional expertise in addiction medicine. The clinic operated on two half days a week on Monday and Wednesday from 9:00 am to 12:30 pm. Patients were seen through a walk-in model so they could engage in treatment when they were ready and motivated [25, 26]. The model gave patients more flexibility as no appointment was required [13]. Although appointments were not scheduled, patients were encouraged to return to the clinic for regular follow-up visits.

At the first visit, patients completed an intake process with the addictions nurse. A detailed history of substance use, motivation for seeking treatment, treatment goals and goals with regards to substance use were discussed and documented. Patients were also seen by the staff physician. An appointment at the clinic included brief counselling and prescription of medications for SUD when indicated. Whenever possible, patients were connected to community support programs and longitudinal counselling services to support their recovery. For OUD, only buprenorphine pharmacotherapy was offered due to the limited hours of operation and variability of expertise among the physicians. In Ontario, physicians do not require a license to prescribe buprenorphine unlike in the United States where an exemption is required. Buprenorphine is recommended as first-line treatment for opioid use disorder in the most recent guidelines [3].

The primary goal of the clinic was not to provide long-term treatment for substance use disorder but rather to initiate treatment when the patient was most motivated to address their disorder. Whenever possible, once the patient’s symptoms were well managed, the patient was transitioned to their primary care provider with an established treatment plan. As SUD is a chronic condition, the patient was encouraged to return to the RAAM clinic if they required additional support.

### Analytic assessment

Data were first analyzed with descriptive statistics using proportions for categorical variables. The Shapiro–Wilk test was used to detect the presence of non-normality. For data with non-normal distributions, descriptive statistics were expressed as median (Mdn) and interquartile range (IQR). Since the data did not follow a normal distribution, the Wilcoxon rank-sum test was used to compare non-parametric paired data from the initial visit and the most recent visit available at the time of the study. All statistical analyses were completed using R [27]. Figures were designed using GraphPad Prism version 4.00 [28].

## Results

### Patient Demographic Information & Substance use Patterns

Over the first 26 weeks, 64 unique patients were seen at the RAAM Clinic at Toronto Western Hospital. Patient demographic information is outlined in Table 1. The median age was 40 (range 20–69) and the population was predominantly male (61%, n = 39). More than half of the patients were referred by primary care providers (55%, n = 35), followed by the emergency department (30%, n = 19) and a withdrawal management service (11%, n = 7). The referral source for 3 patients could not be determined (5%). The most common substance use disorder was AUD (66%, n = 42), followed by OUD (39%, n = 25) and stimulant use disorder (20%, n = 13). The number of substance use disorders diagnosed per patient ranged from 1 to 4, with a mean of 1.28. Other substance use disorders including benzodiazepine use disorder and cannabis use disorder were less common (3%, n = 2).

**Table 1:** Characteristics of patients attending the RAAM Clinic at TWH in the first 26 weeks (*n* = 64)

Characteristic	N	%
Gender		
Male	39	61
Female	25	39
Age		
20–29	13	20
30–39	19	30
40–49	12	19
50–59	14	22
60–69	6	9
Referral Source		
Emergency Department	19	30
Primary Care Provider	35	55
Withdrawal Management Service	7	11
Unknown	3	5
Substance Use Disorders by presentation		
Alcohol use disorder	32	50
Opioid use disorder	13	20
Comorbid opioid use disorder and alcohol use disorders	5	8
Stimulant use disorder	4	6
Comorbid alcohol use disorder and stimulant use disorders	2	3
Comorbid opioid use disorder and stimulant use disorders	4	6
Comorbid alcohol, opioid and stimulant use disorders	3	5
Other	1	1
Substance Use Disorders, Cumulative^a^		
Alcohol use disorder	42	66
Opioid use disorder	25	39
Stimulant use disorder	13	20
Benzodiazepine use disorder	1	1.5
Cannabis use disorder	1	1.5

^a^ Some patients have more than one SUD, counted separately. Percentage expressed as a fraction of total patient population

### Patient retention

Of all patients who completed an intake visit (n = 64), 42% (n = 27) remained on-going patients, 23% (n = 15) were discharged and 34% (n = 22) were lost to follow-up at the end of the study period (Table 2). Of all individuals with AUD who attended the RAAM clinic, 50% (n = 21) remained on-going patients, 14% (n = 6) were discharged to another care provider, and 36% (n = 15) were lost to follow-up. Of all individuals who attended with OUD, 36% (n = 9) remained on-going patients, 32% (n = 8) were discharged to another care provider and 32% (*n* = 8) were lost to follow-up. During the study period, patients attended the clinic an average of 3 times (range: 1–16 visits). The average time between the initial and most recent visit was 3.5 weeks (range: 0–20 weeks).

**Table 2:** Current status of patients by substance use disorder and referral source

Characteristic	On-going Patient		Discharged		Lost to follow-up	
	N	%	N	%	N	%
All Patients	27	42	15	23	22	35
Substance Use Disorder						
Alcohol Use Disorder (*n* = 42)	21	50	6	14	15	36
Opioid Use Disorder (*n* = 25)	9	36	8	32	8	32
Referral Source						
Emergency Department (*n* = 19)	10	53	2	11	7	37
Primary Care Provider (*n* = 35)	13	37	11	31	11	31
Withdrawal Management Service (*n* = 7)	3	43	2	29	2	29

### Prescription of pharmacotherapy

**Fig. 1:**
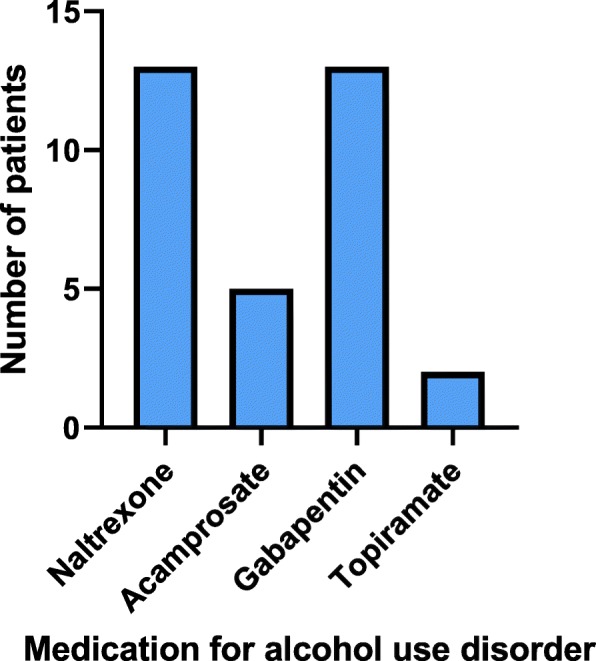
Frequency of medications prescribed for alcohol use disorder for on-going patients and patients discharged to other care settings

Almost all AUD patients who were retained in care (*n* = 27) (remained either on-going patients or were discharged to another care provider) were prescribed medication (93%, n = 25), with a total of 33 prescriptions (Fig. 1). Gabapentin (39%, n = 13) and naltrexone (39%, *n* = 13) were prescribed most frequently, followed by acamprosate (15%, n = 5) and topiramate (6%, n = 2).

Most OUD patients who were retained in care (n = 17) were prescribed buprenorphine (65%, n = 11). Those who were not prescribed buprenorphine declined treatment (24%, n = 4), or prioritized treatment for another substance use disorder (12%, n = 2).

### Alcohol use outcomes

**Fig. 2:**
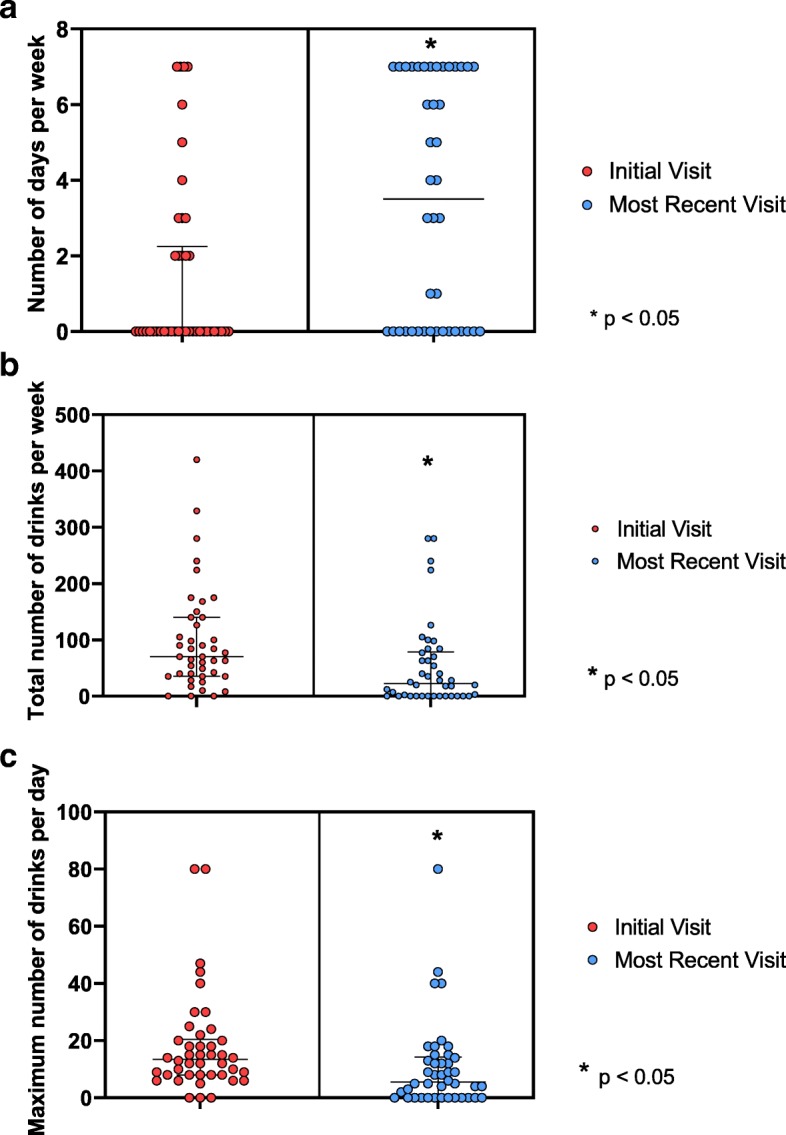
**a**, **b**, **c** Self-reported measures of alcohol consumption for all patients at their initial and most recent visit. 2A: Median and inner quartile range of number of days abstinent per week for all patients at initial visit and at the most recent visit; 2B: Median and inner quartile range of total weekly alcohol consumption for all patients at their initial visit vs. most recent visit; 2C Median and inner quartile range of daily maximum alcohol consumption for all patients at their initial visit and most recent visit

The number of self-reported abstinent days per week in AUD patients (n = 42) increased significantly (Wilcoxon z = − 3.56, p < 0.001) from the initial visit (Median [Mdn] = 0, Interquartile range [IQR] = 0–2) to the most recent visit (Mdn = 3.5, IQR = 0–7, Fig. 2a). Alcohol intake, as defined by the median total weekly consumption for all AUD patients, was significantly lower (z = − 3.89, p < 0.001) at the most recent visit (Mdn = 22.5, IQR = 0–75) compared with the initial visit (Mdn = 70, IQR = 36–137) (Fig. 2b). Additionally, the median maximum number of drinks in a day was significantly lower (z = − 4.03, p < 0.001) at the most recent visit (Mdn = 5.5, IQR = 0–14) than at the initial visit (Mdn = 13.5, IQR = 8–20) (Fig. 2b).

**Fig. 3:**
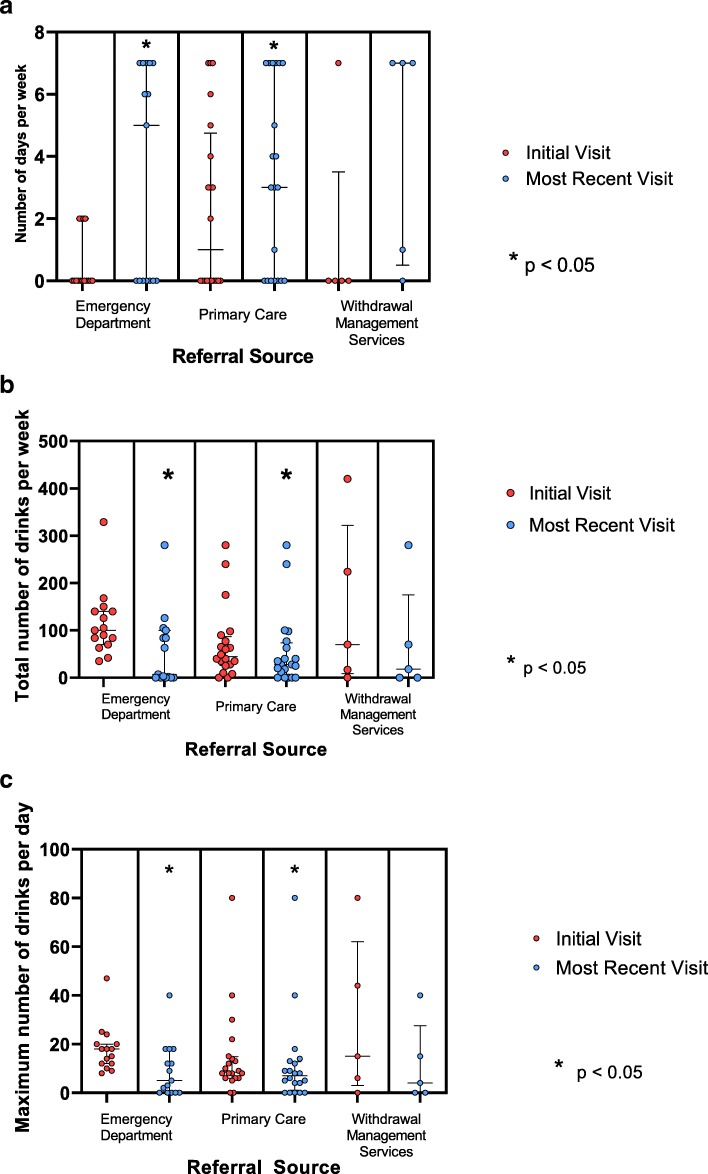
**a**, **b**, **c** Self-reported measures of alcohol consumption for all patients at their initial and most recent visit, by referral source. 3A: Median and inner quartile range of number of days abstinent per week for all patients at the initial visit and the most recent visit, by referral source; 3B: Median and inner quartile range of total weekly alcohol for all patients at the initial visit and the most recent visit, by referral source; 3C: Median and inner quartile range of daily maximum alcohol consumption for all patients at initial visit and the most recent visit, by referral source

Emergency department-referred patients (n = 15) showed a significant increase (Wilcoxon z = − 2.51, p = 0.012) in abstinent days per week from the time of their intake visit (Mdn = 0, IQR = 0–1) to their most recent visit (Mdn = 5, IQR = 0–7, Fig. 3a). Total weekly alcohol consumption for ED-referred patients significantly decreased (z = − 2.61, p = 0.0091) from intake visit (Mdn = 100, IQR = 77–140) at the most recent visit (Mdn = 7, IQR = 0–92, Fig. 3b). Additionally, maximum daily consumption was significantly lower (z = − 2.61, *p* = 0.009) at the most recent visit (Mdn = 5, IQR = 0–15) compared with the initial visit (Mdn = 18, IQR = 12–20, Fig. 3c).

A significant increase in abstinent days per week (Wilcoxon z = − 2.03, p = 0.042) was observed in primary care-referred patients (n = 20), from the intake visit (Mdn = 1, IQR = 0–4) to the most recent visit (Mdn = 3.5, IQR = 0–7, Fig. 3a). Total weekly alcohol consumption significantly decreased (z = − 2.49, p = 0.013) from intake visit (Mdn = 44.5, IQR = 27–80) to their most recent visit (Mdn = 26.5, IQR = 0–46, Fig. 3b). Additionally, maximum daily consumption was significantly lower (z = − 2.62, p = 0.0088) at the time of the most recent visit (Mdn = 5.5, IQR = 0–10) compared with the initial visit (Mdn = 8.5, IQR = 6–14, Fig. 3c).

Patients referred from withdrawal management services (n = 5) did not show a significant increase in abstinent days per week (Wilcoxon z = − 0.89, p = 0.37) from the time of their intake visit (Mdn = 0, IQR = 0–0) to their most recent visit (Mdn = 1, IQR = 0–7, Fig. 3a). Total weekly alcohol consumption did not decrease significantly in patients from withdrawal management services from intake visit (Mdn = 70, IQR = 17–224) to most recent visit (Mdn = 18, IQR = 0–70, Fig. 3b). Additionally, maximum daily consumption did not decrease significantly (z = − 0.89, *p* = 0.37) from the initial visit (Mdn = 15, IQR = 6–44) to the time of the most recent visit (Mdn = 4, IQR = 0–15, Fig. 3c).

### Opioid use outcomes

Amongst individuals who presented with OUD who were retained in care (n = 17), 65% (n = 11) had a negative urine screen for opioids at their most recent visit. A urine test was not done for one patient at their most recent visit (6%, *n* = 1) and 29% (n = 5) had a positive urine test for opioids.

## Discussion

The UHN RAAM clinic was established as part of a wider network of clinics through the province of Ontario to improve access to medical treatment for substance use through a government-funded multi-site pilot which was subsequently expanded provincially [19]. The most common substance use disorder treated at the clinic was AUD, followed by OUD, a reflection of the on-going burden of AUD, the most common substance use disorder in Canada [1], and the rapidly increasing prevalence of OUD, estimated to account for the third highest burden of disease attributable to substance use in Canada after tobacco and alcohol [29].

This study found that the clinic successfully enabled access to evidence-based medications for AUD which have been shown to be under-prescribed in Ontario [30]. The clinic also facilitated opioid agonist therapy of buprenorphine for OUD patients, which is now recommended as the first-line medication for OUD in Canada [31]. The need for improving access to opioid agonist treatment is well-described [32].

Most patients attending the RAAM clinic were referred by primary care providers. Some studies have found that patients with SUD in primary care are infrequently referred to medical supports for substance use and do not have their needs adequately met in primary care settings [13, 14]. Our findings demonstrate that the RAAM model is a feasible and promising specialist consultation service for primary care providers that supports their care for patients with SUD. The RAAM clinic worked closely with the UHN-affiliated Family Health Team (FHT), and this in combination with a shared electronic medical record likely facilitated patient engagement in treatment. In many cases, patients referred from primary care were able to be discharged from the RAAM clinic after the development of a treatment plan and short-term follow-up. The model also allowed for electronic consultation and patients could return without an appointment or referral which ultimately assisted in the reduction of barriers to accessing care.

Patients connected to the clinic from the ED also demonstrated promising treatment outcomes in the RAAM model. Patients who attend the ED for substance use-related concerns are often complex with a high comorbidity of other SUDs and psychiatric conditions [33]. In our study, these patients demonstrated significant reductions in alcohol use when stratified by referral source, likely enabled by the initiation of medical treatment during a crucial window of opportunity when they were motivated to address substance use. This result is consistent with literature showing reduced alcohol consumption following alcohol-related hospitalization as a result of greater awareness of the negative consequences, and the physical and mental distress of the event [20, 34]. Given that many with SUDs frequently use emergency medical services to address their primary care needs [14], the RAAM clinic may be an important source of outpatient medical care for these individuals. The relatively high rate of on-going patients and low rate of discharged patients who were connected through the ED may have been driven by inconsistent access to primary care amongst this sample as well as more severe substance use disorder at intake requiring prolonged stabilization.

The UHN RAAM clinic demonstrated early successes in retention, with 74% of patients with AUD and 68% of patients with OUD retained in care over the 26-week period of this study. In context, a recent study found a 6-month treatment retention rate of 37% for patients with buprenorphine induction in the ED with rapid access to an outpatient community-based addictions clinic [35]. Another study reporting on adult outpatient substance use treatment programs had a completion rate of 40% in the outpatient alcohol treatment program, while the outpatient drug treatment program had a completion rate of 33% [36]. We expect that loss to follow-up will increase over longer durations of treatment; patients entered treatment at variable points throughout the study period. Since previous studies suggest that medication alone may be insufficient [21, 37, 38],patients seen at the RAAM clinic for OUD are connected to counselling supports if they are interested to facilitate a more sustainable recovery. While the impact of this practice could not yet be evaluated, previous studies have not been able to demonstrate that counselling improves long-term treatment retention or prevents relapse for OUD patients [37, 38].

Almost two-thirds of OUD patients who were retained in care remained on buprenorphine treatment at their most recent visit. Previous research has suggested that next day follow-up and a structured prescription regimen such as that utilized in the RAAM clinic may contribute to higher retention rates on buprenorphine [21, 38]. Similarly, two-thirds of this group had negative urine tests for opioids, suggesting that treatment was effective in reducing opioid use.

### Limitations

There are important limitations of this evaluation of the RAAM model in the early stages of its operation. A small patient sample was used for this study which limits the generalizability of the findings. Furthermore, the clinic was staffed by only three physicians and two nurses. The direct impact of their clinical care on treatment outcomes was unable to be isolated from the impact of the model of care and the medications prescribed.

The long-term impact of the RAAM model has yet to be evaluated. Many patients at the clinic have factors associated with an increased risk of relapse for alcohol use disorder over time including cravings, high alcohol intake and impaired control over use [39] and other studies have found that patients on buprenorphine treatment have a higher rate of treatment failure over time than methadone due to a less structured regimen early in treatment [21], a factor that has been addressed in the RAAM model. Therefore, study of a larger patient sample over a longer period is important to better characterize patient outcomes. Evaluation of 3-month and 6-month retention in treatment would allow for better comparisons with other studies [35, 37]. Ideally, evaluation should also be extended beyond when active treatment has concluded [40].

Data for this study was collected for quality improvement purposes via the electronic medical record; patient or provider perspectives were not systematically assessed. Additionally, no comparison of effectiveness using a control group was carried out. Patients who were referred to the RAAM clinic who did not show up for an intake visit could not be tracked centrally; it was therefore not possible to identify how many referred patients chose not to attend the clinic for an initial visit. Tracking these data could allow for a more precise evaluation of treatment retention and inform future initiatives to improve engagement. Similarly, information on patients who were discharged from the clinic could not be tracked, and their substance use patterns after discharge could not be evaluated.

Beyond the need to explore long-term treatment retention and patient outcomes after conclusion of treatment, future studies should explore cost-effectiveness of this treatment model to further support its uptake. Finally, patient needs and experiences in the RAAM model should be explored as previous studies have noted that treatment strategies tailored to patient characteristics (e.g. gender, age, cultural background) rather than type of SUD increase treatment retention [41].

## Conclusion

A new Rapid Access to Addiction Medicine clinic in its first 26 weeks of operation enabled timely access to evidence-based medical treatment for substance use disorder for patients connected through the ED, primary care and withdrawal management services, and facilitated referral to long-term treatment for substance use. This paper describes the early successes of the low-barrier outpatient addiction clinic model in addressing an unmet need in substance use treatment. Patients attending the clinic demonstrated a reduction in alcohol intake and increased opioid abstinence. This study demonstrates that a novel clinical model can increase access to medical care for SUDs and improve outcomes for people with this common but undertreated condition.

## Data Availability

The datasets generated and/or analysed during the current study are not publicly available due to patient confidentiality but are available from the corresponding author on reasonable request.

## Abbreviations

AUD: Alcohol use disorder
ED: Emergency Department
FHT: Family Health Team
IQR: Interquartile Range
Mdn: Median
OUD: Opioid use disorder
RAAM: Rapid access to addiction medicine
SUD: Substance use disorder
UHN: University Health Network
